# Molecular Profiling of Tumor Tissue in Mexican Patients with Colorectal Cancer

**DOI:** 10.3390/cimb44080258

**Published:** 2022-08-20

**Authors:** Beatriz Armida Flores-López, María de la Luz Ayala-Madrigal, José Miguel Moreno-Ortiz, Jorge Peregrina-Sandoval, Miguel Ángel Trujillo-Rojas, José Luis Venegas-Rodríguez, Rosario Hernández-Ramírez, Martha Alejandra Fernández-Galindo, Melva Gutiérrez-Angulo

**Affiliations:** 1 Departamento de Biología Molecular y Genómica, Doctorado en Genética Humana e Instituto de Genética Humana “Dr. Enrique Corona Rivera”, Centro Universitario de Ciencias de la Salud, Universidad de Guadalajara, Guadalajara 44340, Jalisco, Mexico; 2 Departamento de Biología Celular y Molecular, Centro Universitario de Ciencias Biológicas y Agropecuarias, Universidad de Guadalajara, Guadalajara 45200, Jalisco, Mexico; 3 Departamento de Ciencias de la Salud, Centro Universitario de los Altos, Universidad de Guadalajara, Guadalajara 44340, Jalisco, Mexico

**Keywords:** colorectal cancer, massive parallel sequencing, pathogenic variant, likely pathogenic variant, somatic variants, exome sequencing, genetics, molecular pathways

## Abstract

Colorectal cancer is a heterogeneous disease with multiple genomic changes that influence the clinical management of patients; thus, the search for new molecular targets remains necessary. The aim of this study was to identify genetic variants in tumor tissues from Mexican patients with colorectal cancer, using massive parallel sequencing. A total of 4813 genes were analyzed in tumoral DNA from colorectal cancer patients, using the TruSight One Sequencing panel. From these, 192 variants with clinical associations were found distributed in 168 different genes, of which 46 variants had not been previous reported in the literature or databases, although genes harboring those variants had already been described in colorectal cancer. Enrichment analysis of the affected genes was performed using Reactome software; pathway over-representation showed significance for disease, signal transduction, and immune system subsets in all patients, while exclusive subsets such as DNA repair, autophagy, and RNA metabolism were also found. Those characteristics, whether individual or shared, could give tumors specific capabilities for survival, aggressiveness, or response to treatment. Our results can be useful for future investigations targeting specific characteristics of tumors in colorectal cancer patients. The identification of exclusive or common pathways in colorectal cancer patients could be important for better diagnosis and personalized cancer treatment.

## 1. Introduction

Colorectal cancer (CRC) is the third most common cancer in the world, and the second most frequent cause of death due to cancer globally [1]. CRC arises from an accumulation of genetic and epigenetic variations in the intestine epithelium, favoring the transformation of normal mucosa to invasive carcinoma [2]. Sporadic CRC is the main form of presentation for the disease (75%), and is characterized by somatic mutations in the *APC*, *KRAS*, *TP53*, and *BRAF* genes, mainly related to the WNT and EGFR pathways. On the other hand, only 5% of CRC cases are due to a hereditary predisposing syndrome, the most frequent being Lynch Syndrome (LS) and Familial Adenomatous Polyposis (FAP), characterized by loss of function of mismatch repair genes (MMR) and *APC* (WNT pathway), respectively [3]. Advances in human genomics in recent decades have highlighted the importance of CRC genetics in clinical applications [4], with massive parallel sequencing allowing analysis of multiples genes (even the complete genome) in a single assay. Thus, the molecular profiling generated by use of this technique with CRC patients can be fundamental for diagnosis, prognosis, and prediction of response to therapy [5]. In this study, we characterized the pathogenic and likely pathogenic variants screened by an exonic panel that targeted 4813 genes in four tumoral sample tissues, to explore the molecular profile of sporadic and hereditary CRC.

## 2. Materials and Methods

### 2.1. Sample Collection and DNA Extraction

After surgical resection and previous informed consent, tumor tissues from four patients with CRC, confirmed by histopathology, were collected at the Civil Hospital of Guadalajara “Dr. Juan I. Menchaca”. None of the patients had undergone radiation or chemotherapy at the time of collection. The study protocol was approved by the local ethics committee (protocol ID: CI-01417).

Fresh tissue (25–50 mg) was digested with proteinase K overnight at 37 °C; then, DNA was extracted with the “High Pure PCR Template Preparation Kit” (Product number: 11796828001; Roche Diagnostic GmbH, Mannheim, Germany), its concentration and purity was measured with a Qubit 3.0 fluorometer (Life Technologies), and DNA integrity was evaluated on a 1.5% agarose gel stained with GelRed (Catalog number: 41001, Biotium).

### 2.2. Library Preparation and Targeted Sequencing

DNA was adjusted to a final concentration of 4 nM, and library preparation was undertaken with the TruSight One Sequencing panel kit (Catalog number: FC-141-1006, Illumina, San Diego, CA, USA), targeting the exonic regions of 4813 genes. Genomic DNA tagmentation, clean-up, tagmented DNA amplification, probe hybridization and capture, enrichment, enriched library amplification, and library cleanup was performed following manufacturer’s instructions. The generated library was sequenced on the Illumina MiSeq in accordance with manufacturer’s instructions.

### 2.3. Data Analysis

Data obtained from the sequencing reaction was aligned to the NCBI 37/hg19 genome reference to create the .bam file on the MiSeq, and then exported to BaseSpace (Illumina) for variant identification and generation of the .vcf file. BaseSpace Variant Interpreter software (Illumina) (accessed on June 2020) was used for data analysis. Filters were applied for variant selection quality, including passing filter and alt allele depth >30%, and only variants with pathogenic and likely pathogenic clinical association were chosen. The depth average for the selected variants was 143X.

Genes in CRC patients harboring variants were listed and pathway enrichment analysis performed in the Reactome software (https://reactome.org/ accessed on 20 June 2022), a Voronoi diagram was used for visualization of over-represented pathways [6].

## 3. Results

### 3.1. Patient Characterization

Four unrelated patients with histopathological diagnoses of CRC were included; all patients were from Jalisco state in Mexico. Clinicopathological and demographic characteristics are shown in Table 1. Patient one mentioned drug habits including alcohol, tobacco, marijuana, methamphetamine, and cocaine for five years before CRC diagnosis. No family history was reported in the patients, however, considering the molecular results for patient one (discussed later in this paper), he was suspected of Lynch syndrome. Moreover, during the colonoscopy procedure multiple polyps were identified for patient two, and total colectomy was performed, therefore this patient was clinically diagnosed with FAP.

### 3.2. Variant Identification in Tumor Samples

To identify tumor variants in colorectal tissue, an exonic panel of 4813 genes was utilized for massive parallel sequencing. We detected an average of 8703 variants (ranging from 7754 to 9734 variants). A total of 192 pathogenic or likely pathogenic variants were chosen for characterization, based on clinical significance reported by the Variant Interpreter platform. Among the variants, 60% were indels (115/192) and the remaining 40% were single nucleotide variants (SNV) (77/192) (Table 2), of which 43% were missense (33/77), 40% nonsense (31/77), and 17% were at the splicing site (13/77). All genes had already been reported as affected in CRC, according to the COSMIC database, although 46 variants were not reflected in any previous report and were considered novel variants. More information about each variant can be found in Supplementary Material File S1. The sequence data reported in this paper were deposited in the Sequence Read Archive (SRA) platform operated by the National Center for Biotechnology Information (NCBI), the European Bioinformatics Institute (EBI) and the DNA Data Bank of Japan (DDBJ), with the BioProject accession numberPRJNA78898 [7]. Sequence data are available at https://www.ncbi.nlm.nih.gov/sra/.

### 3.3. Data Processing

The 192 variants were distributed in 176 genes, and of these 136 were in patient one (Table 3). An enrichment analysis was performed individually in the Reactome database for each patient, and pathway over-representation per patient was observed in the Voronoi diagram (Figure S1). Some genes could not be included in the pathway analysis as they were absent in Reactome database, although different alias were introduced during the analysis. For patient one, 22 identifiers were absent in Reactome (*ANXA11, BCORL1, CCDC40, CCN6, CHD2, DNAI2, DTNA, GSE1, HAX1, HPS6, HYDIN, LMTK3, MAP7D3, MTUS1, PTPN21, RNASEH2B, RSPH4A, SETX, TBC1D23, TOP1MT, ZC3H3, ZNF469*), three could not be identified for patient two (*HYDIN, PPP2R2B* and *TMPRSS5*), in patient three there were four (*A4GALT, PKHD1, PRF1* and *ROPN1L*), and two identifiers were absent in patient four (*HYDIN* and *PTCD1*).

**Figure 1:**
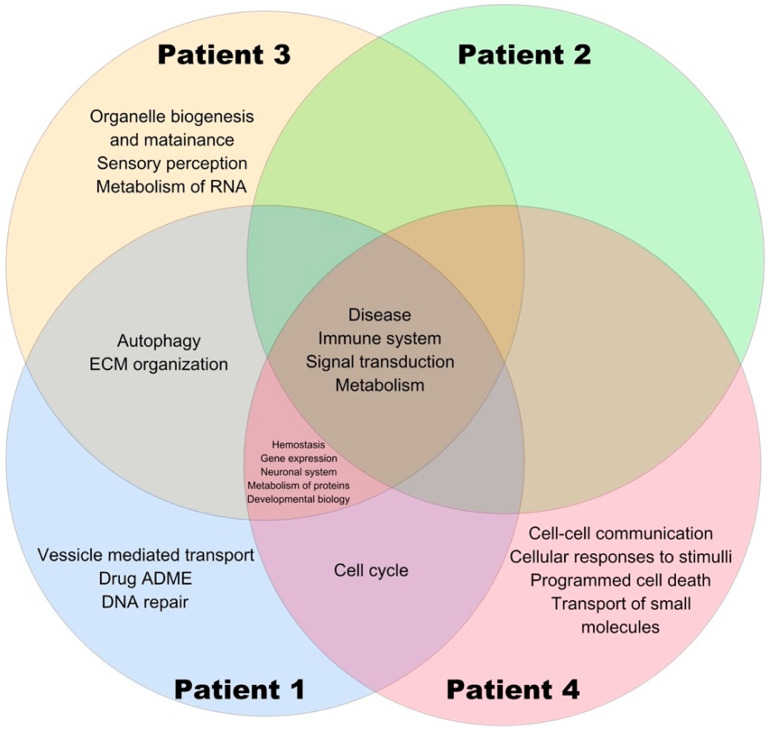
Venn diagram for pathways in the colorectal cancer patients.

The Venn diagram showed overlaps of pathways in patients included in the study (Figure 1). Disease, signal transduction, metabolism, and immune system pathways were common in the four patients, although some were more over-represented than others. Moreover, exclusive pathways were found in three patients: DNA repair, drug ADME, and vesicle-mediated transport in patient one; organelle biosynthesis and maintenance, autophagy, and metabolism of RNA in patient three; and cellular responses to stimuli, cell-cell communication, transport of small molecules, and programmed cell death in patient four.

## 4. Discussion

Colorectal cancer development is characterized by the successive accumulation of genetic alterations that activate protooncogenes and inactivate tumor suppressor genes [4]. In this study, analysis with a next generation sequencing panel of four tumor DNA samples from patients with CRC showed a total of 192 variants with pathogenic and likely pathogenic clinical significance, 146 of which were found in the youngest patient (patient one, 26 yrs).

Pathway enrichment analysis in the Reactome database revealed that disease, signal transduction, and immune system were common pathways in the four patients. Disease and signal transduction pathways were over-represented and interconnected in these patients, mainly by alterations in cancer-associated genes. Within these pathways, patient one showed an evident and exclusive over-representation of DNA repair, caused by alterations in *MSH3*, *MSH6*, *ATM*, and *RBBP8* genes. *MSH3* and *MSH6* genes are involved in mismatch DNA repair (MMR), while *ATM* and *RBBP8* are functional in double-strand break repair. Although no familial history was reported in this patient, two Bethesda criteria for Lynch syndrome were met, CRC diagnosis under 50 years old and microsatellite instability status (data not shown), which correlated with the identified variants in MMR genes. Win et al. in 2011 found 2.3% of de novo mutations in 261 probands with LS [8], therefore, we do not exclude the possibility that de novo events occurred in our patient. In addition, inside the disease pathway, a subset of signaling by WNT in cancer was found in patients one and two, however, the over-representation of WTN was notable in patient one, who had mutations in several genes that interact in the pathway, in contrast to the female with FAP (patient two) who harbored only one variant in *APC*. On the other hand, the subset of EGFR signaling in cancer was clearly over-represented in patients three and four, both showing mutated *KRAS*, and *PIK3CA* was also affected in the oldest patient (patient four).

According to this analysis, the immune system was involved in all four patients, and the main affected genes were related to the complement system and NK cells. The enrichment analysis showed that the complement system and the NK cells were altered in the youngest patient (patient one) with the identifiers *CFI*, *FCN3*, *KIR3DL1* and *KIR2DL4. KIR2DL4* was also affected in those with sporadic CRC (patients three and four), while in the FAP patient (patient two) the altered gene involved in the complement system was *C8B*. Variants found in immune system genes can produce truncated proteins, either nonsense or indel mutations, therefore, tumor cell clearance could be affected in these patients. However, several factors can combine to determine tumor induction or repression within the immune system, for example, the type or characteristics of the cancer and the combination of affected genes [9,10].

Notwithstanding exclusive pathways found in three patients, it is important to note that some of those identified are relevant for development, progression, and/or treatment response in cancer. For example, alterations in genes associated with drug metabolism such as *ABCC4* and *NAT1* found in the youngest patient (patient one) could modulate response to treatment. The transporter protein encoded by the *ABCC4* gene is implicated in resistance to 5-Fluorouracil in pancreatic cells (https://www.pharmgkb.org/ accessed on 22 June 2022), a principal drug employed in colorectal cancer treatment, and although the protein produced by the *NAT1* gene is not involved in metabolism of colorectal cancer chemotherapy, it participates in the mesalazine catabolism pathway (https://www.pharmgkb.org/ accessed on 22 June 2022). Mesazaline is a drug employed in inflammatory bowel disease treatment, and has been evaluated in animal models for Lynch syndrome due anti-inflammatory and chemopreventive properties [11]. Moreover, Zhu et al. (2021) reported copy number loss of *NAT1* in gastric and colorectal cancers [12].

In the autophagy subset, particular mitophagy alterations were found in a patient with sporadic CRC (patient three) where the *PRKN* gene was affected. The role of mitophagy in cancer is unclear, but failure in this mechanism is favorable for tumor development [13]. PRKN has a major role in mitophagy regulation, and its loss has been reported in 33% of CRC cases. Evidence also suggests that loss of *PRKN* function either by deletions or pathogenic mutations may be involved in tumor progression and metastasis, consistent with the patient’s stage in this study [14]. Additionally, this patient showed alterations in RNA metabolism, and the affected gene was *ADAR* [15]. This gene encodes an adenosine deaminase protein involved in the conversion of adenosines to iosines during RNA editing; thus, mutations in ADAR lead to unedited RNA and consequently could alter the structure and function of its proteins. This variant has been described only in melanoma [16,17], but its effect on protein structure and function is unknown. However, a mutation with gain of function could explain its role in cancer, since overexpression and other mutations have been described in cancer of the large intestine [16,18].

In the oldest patient included in this study, cellular responses to stimuli and programmed cell death pathways were related to the *TP53* gene, a tumor suppressor affected in up to 60% of sporadic CRC patients and related to the classical adenoma–carcinoma sequence. *TP53* is considered “the guardian of the genome”, given its function as a key regulator in cellular growth and gene transcription [19]. It is also important to highlight the need of identifying *TP53* somatic mutations in CRC patients, since it has been shown that tumors could be resistant to classical chemotherapies, and some authors have proposed that *TP53* mutants are a suitable target for immunotherapies [20].

Several studies [21,22,23,24] have been carried out involving massive parallel sequencing in CRC patients, and the main pathways associated were WNT, MAPK, TGFβ, and PI3K. The whole exome analysis performed by The Cancer Genome Atlas Network in 2012 [21] found alterations mainly in *APC*, *TCF7L2*, *PIK3CA*, and *KRAS* genes; however, high frequencies of mutated variants in *ATM*, *ARID1A*, *TP53*, *MSH3*, *MSH6*, and *SLC9A9* genes were reported in the present study. In 2018, Sanchez-Vega et al. analyzed 33 cancer types including CRC and found high frequencies of variants in WNT, PI3K, TGFβ, RAS, HIPP, MYC, NOTCH, TP53, cell cycle, and NRF2 pathways, although the last two were less frequently mutated [22]. Notwithstanding variants in additional genes that were found in those studies, our work highlights not only the classical pathways shared by CRC patients, but also exclusive pathways that could predict the prognosis of the disease or treatment response.

The MMR deficiency found in the youngest patient (patient one) could be associated with the increase of variants, mainly those classified as indels (101 of 146), which were not observed in the other patients reported in this study. This patient was the individual with the greatest history of risk factors; among those mentioned by the patient were tobacco, alcohol, marijuana, cocaine, and methamphetamine, substances are known to produce cellular and genetic damage [25]. DNA repair mechanisms could repair some defects caused by carcinogens, but in this patient multiple repair pathways were affected by pathogenic variants, which could cause a negative feedback loop of DNA damage accumulation. Xicola et al. (2020) hypothesized that Lynch-like presentation in patients with genomic instability could be the result of mutations in genes that maintain genome integrity, such as *REV3L*, which encodes for the catalytic subunit of polymerase polζ [26]. Patient one also harbored a mutation in *REV3L*, but germline variants could not be ruled out. Moreover, the analysis of *MLH1* methylation was negative (data not shown), so sporadic CRC could be excluded. However, in addition to de novo LS, a Lynch-like syndrome could be an alternative suspected form of hereditary CRC in this patient.

The analyzed patients harbored tumors located mainly in the rectum (patients two, three, and four) and one patient had a left-sided tumor. Despite diversity in patient characteristics, recent studies have demonstrated that left colon and rectum cancers can be treated as a single entity because of similarities in incidence, genetic aspects, and embryological origin [27]. However, inter- and intratumoral molecular heterogeneities have also been described in CRC [28].

The results for patient with probable LS (patient one) showed the most significant findings in the enrichment analysis compared to the FAP patient (patient two). In hereditary CRC patients, it is important to considered that loss of function in MMR genes can lead to an accumulation of mutations and errors in regions rich in microsatellite-type repeats. Thus, some variants found in patient one could be derived from defects in repair systems and not exactly because of relevance in cancer development [29]. On the other hand, differences in pathways were also found between the sporadic cases (patients three and four), as described in previous paragraphs and evident in the Venn diagram. This highlights the wide heterogeneity in sporadic CRC and the importance of analyzing each patient.

The main limitations of this study were the sample size and the lack of peripheral blood to screen the germinal variants to determine which were a product of the carcinogenic process. However, any change in the genome could increase tumor development in these patients, regardless whether it was somatic or germline.

In conclusion, integrative analysis of the exome sequencing data of CRC tissues provides valuable information about affected pathways in CRC, and allows analysis of the broad scenery of CRC pathogenesis. Analysis of a great number of patients will empower these results and give accurate information about the study population. This approach to somatic variants can help to drive the management and treatment of patients according to their molecular tumor characteristics, which directly impacts prognosis and life expectancy. Even though our sample size did not allow us to establish a direct relationship between genomic data and clinical characteristics in these patients, knowledge of the different processes and altered genes in our patients has opened up the panorama of possible research targets. In the future, these could be transferred into a clinical setting for diagnosis and management of patients.

## Figures and Tables

**Table 1:** Clinicopathological and demographic characteristics of CRC patients.

Patient	Age	Sex	Tumor Localization	Histology Type	Metastasis	Stage	Diagnosis
1	26	Male	Left colon	Mucinous Adenocarcinoma †	No	II	LS?
2	34	Female	Rectum	Adenocarcinoma †	Node	III	FAP
3	48	Male	Rectum	Adenocarcinoma †	Node	III	Sporadic CRC
4	66	Male	Rectum	Adenocarcinoma ‡	Node	III	Sporadic CRC

Age in years; † G2: moderately differentiated; ‡ G3: Poorly differentiated; CRC: colorectal cancer; LS?: Lynch syndrome suspect; FAP: familial adenomatous polyposis.

**Table 2:** Number and types of variants by type found in colorectal cancer patients.

Patient Number	1	2	3	4	Total
Variant Type					
Pathogenic	22	7	9	2	40
Likely pathogenic	124	9	10	9	152
*Total of variants*	*146 (76%)*	*16 (8%)*	*19 (10%)*	*11 (6%)*	*192 (100%)*
Indels	101	5	6	3	115
SNV	45	11	13	8	77

**Table 3:** Affected genes in the colorectal cancer patients.

Patient 1	Patient 2	Patient 3	Patient 4
ABCB4, ABCC4, ACOX1, ADAMTS18, ADAMTSL2 *, ADAMTSL4, AFP, ALDOA, AMER1, **ANK2**, ANO10, ANXA11, APC *, ARID1A, ATM, **BCO1**, BCORL1, BMPR2, CASP5, CASR, CCDC40, CEL *, **CFI**, CHD2, **COL6A5**, CPZ, **CSNK2A2**, **CUL5**, CYLD, CYP2D6, DCLRE1C, DHX16, **DISP1**, DLGAP3, DNAI2, **DPM1**, **DTNA**, EGR2, EPHA2, **EPHA3** *, FBN2, FBN3, FBXW7, FCN3, FGG, FLCN, **GJA8**, GRK4, GSE1, HAX1, HMBS, HNF1A, HPS6, HTR3C, HTT, HYDIN, IQGAP1, ITPKC, **ITPR1, KAT6B**, KIR2DL4, KIR3DL1, KMT2E, KRAS**, LARS2, LIG3**, LMTK3, LTBP4, MAD1L1, MAP7D3, MASTL**, MIA3**, MLH3, **MOGS**, MSH3, MSH6 *, **MST**1, MTMR9, MTUS1 *, **MUC5B**, MYB, MYH14, MYL2, MYO15A, MYO9B, NAT1, NBAS, NLRP12, NOD2, **NRXN1**, OBSL1, **PCARE**, PCDH15, PHF2 *, PHKB, **PIK3C2G**, **PLEC**, **PLEKHG4**, PRRT2, PRSS12, **PRX**, PTCH1, PTEN, PTPN21, PZP, RBBP8, **REV3L**, RNASEH2B, **ROR2**, **RSPH4A**, SALL4, SCN9A *, SEC63 *, SERPINA6, SETX, **SLC9A9**, **SPTB**, STRA6, **SUCLG1**, TAP2, TBC1D23, **TBX1**, TCF7, TCF7L2, TGFBR2, TGM1, TMPO, TNXB, TOP1MT, TPP2, TRPM1, TUBB2B, CCN6, **ZC3H3**, ZFP90, ZNF469	ABCC6 *, **APC**, *C8B*, ***DOCK4***, *ENO3*, *GALNS*, *HLA-DRB1*, *HYDIN*, PPP2R2B *, SCN9A *, *TMPRSS5*	***A4GALT***, *ADAR*, *ALDOB*, *CNGB1*, ***COL4A3***, ***EYS***, *FBN1*, ***HNF1B***, *KIR2DL4*, *KRAS*, *MC1R*, *PKHD1*, *PRF1*, *PRKN*, *RET*, *ROPN1L*, *SARDH*, *SCO2*, *TRPV4*	*CD109*, *HYDIN*, *KIR2DL4*, *KRAS*, *MS4A2*, *MUC6*, *PAFAH1B3*, *PIK3CA*, *PTCD1*, *TP53*, *TRPV4*
*136 genes*	*11 genes*	*19 genes*	*11 genes*

Genes with novel variants are shown in bold. * Genes with more than one variant in the same patient.

## Data Availability

The authors confirm that the data supporting the findings of this study are available within the article and its supplementary materials. The sequence data are available in the Sequence Read Archive (SRA) platform with accession number PRJNA78898.

## Supplementary Materials

The following supporting information can be downloaded at: https://www.mdpi.com/article/10.3390/cimb44080258/s1, File S1: Variant description per patient. Figure S1: Voronoi diagram providing a pathways overview for the four patients with colorectal cancer.
