# Supplementary material for: Molecular Profiling of Tumor Tissue in Mexican Patients with Colorectal Cancer
Source: Curr Issues Mol Biol. 2022 Aug 20;44(8):3770–8. doi: 10.3390/cimb44080258 (PMC9406459; doi:10.3390/cimb44080258)
Supplement: Supplementary file 1 [file cimb-44-00258-s001.zip › File S1-variant description-Flores-López BA.pdf]

Clinical significance an association was in accordance to data reported up to June 2020, any subsequent changes are not considered.

Gene	Location	Rs / COSMIC ID	Genomic consequence	Type	Protein alteration	Protein consequence	Exon	Clinical significance
ABCB4	chr7:87051452	COSV55938636	c.2301del	Deletion	p.(Phe767LeufsTer30)	Frameshift	18	Likely pathogenic
ABCC4	chr13:95696016	rs756859429	c.3655del	Deletion	p.(Ile1219SerfsTer11)	Frameshift	29	Likely pathogenic
ACOX1	chr17:73945658	COSV53133165	c.1502del	Deletion	p.(Asn501ThrfsTer6)	Frameshift	11	Likely pathogenic
ADAMTS18	chr16:77398193		c.864del	Deletion	p.(Lys288AsnfsTer34)	Frameshift	5	Likely pathogenic
ADAMTSL2	chr9:136419553		c.1018del	Deletion	p.(Gln340SerfsTer21)	Frameshift	10	Likely pathogenic
ADAMTSL2	chr9:136439972		c.2848del	Deletion	p.(His950ThrfsTer?)	Frameshift	19	Likely pathogenic
ADAMTSL4	chr1:150526251		c.789del	Deletion	p.(Ser264HisfsTer6)	Frameshift	6	Likely pathogenic
AFP	chr4:74309135		c.690del	Deletion	p.(Phe230LeufsTer9)	Frameshift	6	Likely pathogenic
ALDOA	chr16:30080978	rs773601711	c.951dup	Duplication	p.(Ala318ArgfsTer14)	Frameshift	8	Likely pathogenic
AMER1	chrX:63412029	COSV57658653	c.1138G>T	Transversion	p.(Glu380Ter)	Nonsense	2	Likely pathogenic
ANK2	chr4:114264256		c.4212del	Deletion	p.(Phe1404LeufsTer28)	Frameshift	34	Likely pathogenic
ANO10	chr3:43647213	rs764901939	c.132del	Deletion	p.(Asp45MetfsTer12)	Frameshift	2	Likely pathogenic
ANXA11	chr10:81928878	rs771435989	c.408del	Deletion	p.(Gly137AspfsTer14)	Frameshift	6	Likely pathogenic
APC	chr5:112151204	rs786201856	c.847C>T	Transition	p.(Arg283Ter)	Nonsense	9	Pathogenic
APC	chr5:112175676	rs387906234	c.4393_4394del	Deletion	p.(Ser1465TrpfsTer3)	Frameshift	16	Pathogenic
ARID1A	chr1:27105931	COSV61371147	c.5548del	Deletion	p.(Asp1850ThrfsTer33)	Frameshift	20	Likely pathogenic
ATM	chr11:108141874	rs587781558	c.2921+1G>A	Transition		Splice region	Intron 20	Pathogenic
BCO1	chr16:81295865		c.448C>T	Transition	p.(Gln150Ter)	Nonsense	4	Likely pathogenic
BCORL1	chrX:129148573	COSV54392560	c.1825C>T	Transition	p.(Arg609Ter)	Nonsense	3	Likely pathogenic
BMPR2	chr2:203420130	rs772920507	c.1748del	Deletion	p.(Asn583ThrfsTer44)	Frameshift	12	Likely pathogenic
PCARE	chr2:29295612		c.1516C>T	Transition	p.(Gln506Ter)	Nonsense	1	Likely pathogenic
CASP5	chr11:104878041	rs755365949	c.241del	Deletion	p.(Thr81GlnfsTer26)	Frameshift	3	Likely pathogenic
CASR	chr3:121973139	rs1450056958	c.108del	Deletion	p.(Leu37SerfsTer8)	Frameshift	2	Likely pathogenic
CCDC40	chr17:78013765	rs397515393	c.248del	Deletion	p.(Ala83ValfsTer84)	Frameshift	3	Pathogenic
CEL	chr9:135946954	rs756449511	c.2082dup	Duplication	p.(Val695ArgfsTer6)	Frameshift	11	Likely pathogenic
CEL	chr9:135946954	rs756449511	c.2081_2082dup	Duplication	p.(Val695ProfsTer13)	Frameshift	11	Likely pathogenic
CFI	chr4:110687710		c.328G>T	Transversion	p.(Gly110Ter)	Nonsense	2	Likely pathogenic
CHD2	chr15:93545434	rs749969667	c.4173del	Deletion	p.(Lys1391AsnfsTer15)	frameshift	33	Likely pathogenic
COL6A5	chr3:130148428		c.5248G>T	Transversion	p.(Glu1750Ter)	Nonsense	32	Likely pathogenic
CPZ	chr4:8603045	rs753969942	c.322del	Deletion	p.(Arg108GlyfsTer90)	Frameshift	3	Likely pathogenic
CSNK2A2	chr16:58200489		c.826C>T	Transition	p.(Gln276Ter)	Nonsense	9	Likely pathogenic
CUL5	chr11:107925659		c.757C>T	Transition	p.(Arg253Ter)	Nonsense	7	Likely pathogenic
CYLD	chr16:50826564	COSV61096477	c.2305dup	Duplication	p.(Ile769AsnfsTer14)	Frameshift	17	Likely pathogenic
CYP2D6	chr22:42524213	rs72549352	c.805dup	Duplication	p.(Arg269ProfsTer5)	Frameshift	5	Likely pathogenic
DCLRE1C	chr10:14968855	rs41298896	c.959C>G	Transversion	p.(Ser320Cys)	Missense	11	Likely pathogenic
DHX16	chr6:30621048	COSV53312568	c.3097del	Deletion	p.(Ile1033Ter)	Frameshift	20	Likely pathogenic
DISP1	chr1:223164922		c.728del	Deletion	p.(Asn243IlefsTer8)	Frameshift	7	Likely pathogenic
DLGAP3	chr1:35370731	rs766293453	c.254del	Deletion	p.(Gly85ValfsTer215)	Frameshift	3	Likely pathogenic
DNAI2	chr17:72308163	rs141581673	c.1516C>T	Transition	p.(Arg506Ter)	Nonsense	12	Pathogenic
DPM1	chr20:49574994		c.67C>T	Transition	p.(Arg23Ter)	Nonsense	1	Likely pathogenic
DTNA	chr18:32395873		c.609del	Deletion	p.(Val204SerfsTer3)	Frameshift	6	Likely pathogenic
EGR2	chr10:64573574	COSV54346522	c.824del	Deletion	p.(Gly275AlafsTer6)	Frameshift	2	Likely pathogenic
EPHA2	chr1:16464489	rs34192549	c.1171G>A	Transition	p.(Gly391Arg)	Missense	5	Likely pathogenic
EPHA3	chr3:89468398		c.1938del	Deletion	p.(Glu647ArgfsTer9	Frameshift	11	Likely pathogenic
EPHA3	chr3:89499363	rs1317589623	c.2538del	Deletion	p.(Met847TrpfsTer10)	Frameshift	15	Likely pathogenic
FBN2	chr5:127610310	rs748600284	c.7659dup	Duplication	p.(Phe2554ValfsTer4)	Frameshift	60	Likely pathogenic
FBN3	chr19:8196625	rs773242834	c.1803del	Deletion	p.(Leu602TrpfsTer3)	Frameshift	15	Likely pathogenic
FBXW7	chr4:153249384	rs1057519895	c.1154G>A	Transition	p.(Arg385His)	Missense	8	Likely pathogenic
FCN3	chr1:27699671	rs532781899	c.349del	Deletion	p.(Leu117SerfsTer65)	Frameshift	5	Pathogenic
FGG	chr4:155530877	rs6063	c.571G>A	Transition	p.(Gly191Arg)	Missense	6	Pathogenic
FLCN	chr17:17119709	rs80338682	c.1285del	Deletion	p.(His429ThrfsTer39)	Frameshift	11	Pathogenic
GJA8	chr1:147380341		c.263dup	Duplication	p.(Ser89ValfsTer33)	Frameshift	2	Likely pathogenic
GRK4	chr4:3015469	rs375862689	c.664dup	Duplication	p.(Arg222LysfsTer8)	Frameshift	8	Likely pathogenic
GSE1	chr16:85682289	rs764524595	c.366dup	Duplication	p.(Val123ArgfsTer17)	Frameshift	3	Likely pathogenic
HAX1	chr1:154246357	rs773524232	c.430del	Deletion	p.(Val144SerfsTer70)	Frameshift	3	Likely pathogenic
HMBS	chr11:118960923	rs118204098	c.446G>A	Transition	p.(Arg149Gln)	Missense	8	Pathogenic
HNF1A	chr12:121432115	rs762703502	c.864del	Deletion	p.(Pro291GlnfsTer51)	Frameshift	4	Pathogenic
HPS6	chr10:103826918	COSV54624914	c.1692del	Deletion	p.(Phe565LeufsTer48)	Frameshift	1	Likely pathogenic
HTR3C	chr3:183777416	rs1394232956	c.919dup	Duplication	p.(Leu307ProfsTer39)	Frameshift	7	Likely pathogenic
HTT	chr4:3162034	rs34315806	c.3785C>T	Transition	p.(Thr1262Met)	Missense	29	Pathogenic
HYDIN	chr16:70896016	rs11337008	c.11712del	Deletion	p.(Gln3905ArgfsTer5)	Frameshift	69	Likely pathogenic
IQGAP1	chr15:91019925	rs771739474	c.2822del	Deletion	p.(Asn941IlefsTer8)	Frameshift	24	Likely pathogenic
ITPKC	chr19:41224076	COSV54573568	c.1041del	Deletion	p.(Ser348ProfsTer45)	Frameshift	1	Likely pathogenic
ITPR1	chr3:4776846		c.5309-2A>G	Transition		Splice region	Intron 43	Likely pathogenic
KAT6B	chr10:76739065		c.2199G>A	Transition	p.(Trp733Ter)	Nonsense	10	Likely pathogenic
KIR2DL4	chr19:55324674	rs11371265	c.810dup	Duplication	p.(Met271AsnfsTer108)	Frameshift	6	Likely pathogenic
KIR3DL1	chr19:55331298	rs777544872	c.487del	Deletion	p.(Asp163ThrfsTer23)	Frameshift	4	Likely pathogenic
KMT2E	chr7:104746985	COSV57571025	c.2619del	Deletion	p.(Lys873AsnfsTer8)	Frameshift	19	Likely pathogenic
KRAS	chr12:25398284	rs121913529	c.35G>A	Transition	p.(Gly12Asp)	Missense	2	Pathogenic
LARS2	chr3:45561768		c.2272G>T	Transversion	p.(Gly758Ter)	Nonsense	19	Likely pathogenic
LIG3	chr17:33318090		c.1002del	Deletion	p.(Phe334LeufsTer12)	Frameshift	5	Likely pathogenic
LMTK3	chr19:48994757	rs758225154	c.4218dup	Duplication	p.(Glu1407ArgfsTer162)	Frameshift	14	Likely pathogenic
LTBP4	chr19:41132910	rs754757253	c.4224dup	Duplication	p.(Ala1409ProfsTer?)	Frameshift	31	Likely pathogenic
MAD1L1	chr7:2265161	rs121908982	c.175C>T	Transition	p.(Arg59Cys)	Missense	4	Pathogenic
MAP7D3	chrX:135314194	COSV60169925	c.922del	Deletion	p.(Gln308ArgfsTer2)	Frameshift	8	Likely pathogenic
MASTL	chr10:27459056	COSV60909589	c.1173del	Deletion	p.(Lys391AsnfsTer12)	Frameshift	8	Likely pathogenic
MIA3	chr1:222823724		c.3720+2T>C	Transversion		Splice region	Intron 9	Likely pathogenic
MLH3	chr14:75514604	rs766244000	c.1755del	Deletion	p.(Glu586AsnfsTer24)	Frameshift	2	Likely pathogenic
MOGS	chr2:74689157		c.1759del	Deletion	p.(Arg587GlyfsTer7)	Frameshift	4	Likely pathogenic
MSH3	chr5:79970915	rs587776701	c.1148del	Deletion	p.(Lys383ArgfsTer32)	Frameshift	7	Pathogenic
MSH6	chr2:48026290	rs753796271	c.1168del	Deletion	p.(Asp390IlefsTer21)	Frameshift	4	Pathogenic
MSH6	chr2:48030639	rs267608087	c.3261dup	Duplication	p.(Phe1088LeufsTer5)	Frameshift	5	Pathogenic
MST1	chr3:49721755		c.2008del	Deletion	p.(Ala670ProfsTer24)	Frameshift	17	Likely pathogenic
MTMR9	chr8:11162509	rs1477512676	c.584del	Deletion	p.(Asn195MetfsTer4)	Frameshift	4	Likely pathogenic
MTUS1	chr8:17513478		c.3001dup	Duplication	p.(Thr1001AsnfsTer8)	Frameshift	9	Likely pathogenic
MTUS1	chr8:17611996		c.1321del	Deletion	p.(Ser441LeufsTer32)	Frameshift	2	Likely pathogenic
MUC5B	chr11:1251256		c.1242G>A	Transition	p.(Trp414Ter)	Nonsense	11	Likely pathogenic
MYB	chr6:135518338	COSV57195899	c.1449del	Deletion	p.(Lys483AsnfsTer8)	Frameshift	10	Likely pathogenic
MYH14	chr19:50747534	rs119103280	c.1150G>T	Transversion	p.(Gly384Cys)	Missense	11	Pathogenic
MYL2	chr12:111348951	rs786205430	c.431del	Deletion	p.(Pro144LeufsTer3)	Frameshift	7	Likely pathogenic
MYO15A	chr17:18025232	COSV52752802	c.3123del	Deletion	p.(Lys1042ArgfsTer16)	Frameshift	2	Likely pathogenic

MYO9B	chr19:17322818	rs1388909247	c.6178del	Deletion	p.(Arg2060GlyfsTer68)	Frameshift	40	Likely pathogenic
NAT1	chr8:18080115	rs5030839	c.745C>T	Transition	p.(Arg249Ter)	Nonsense	6	Likely pathogenic
NBAS	chr2:15616000	rs1460429336	c.1151_1152del	Deletion	p.(Lys384ArgfsTer43)	Frameshift	14	Likely pathogenic
NLRP12	chr19:54313646	rs752493616	c.1266dup	Duplication	p.(Leu423AlafsTer55)	Frameshift	3	Likely pathogenic
NOD2	chr16:50745330	rs754761524	c.1515dup	Duplication	p.(Ser506ValfsTer73)	Frameshift	4	Likely pathogenic
NRXN1	chr2:50318459		c.3838+2T>C	Transition		Splice region	Intron 20	Likely pathogenic
OBSL1	chr2:220420931	rs764560783	c.4420C>T	Transition	p.(Arg1474Ter)	Nonsense	14	Likely pathogenic
PCDH15	chr10:55626401	rs748706627	c.3732+1G>A	Transition		Splice region	Intron 28	Likely pathogenic
PHF2	chr9:96422612	rs775621348	c.1475del	Deletion	p.(Lys492ArgfsTer6)	Frameshift	12	Likely pathogenic
PHF2	chr9:96422612	rs775621348	c.1475dup	Duplication	p.(Thr493AspfsTer21)	Frameshift	12	Likely pathogenic
PHKB	chr16:47683034	rs1336328837	c.1718_1719del	Deletion	p.(Val573GlyfsTer11)	Frameshift	18	Likely pathogenic
PIK3C2G	chr12:18439842		c.745dup	Duplication	p.(Cys249LeufsTer15)	Frameshift	3	Likely pathogenic
PLEC	chr8:145007023		c.2086C>T	Transition	p.(Arg696Ter)	Nonsense	14	Likely pathogenic
PLEKHG4	chr16:67318243		c.1580del	Deletion	p.(Pro527LeufsTer12)	Frameshift	11	Likely pathogenic
PRRT2	chr16:29825012	rs769519069	c.640del	Deletion	p.(Ala214ProfsTer15)	Frameshift	2	Likely pathogenic
PRSS12	chr4:119203243	rs189873467	c.2476G>T	Transversion	p.(Gly826Ter)	Nonsense	13	Likely pathogenic
PRX	chr19:40903511		c.748del	Deletion	p.(Gln250ArgfsTer63)	Frameshift	7	Likely pathogenic
PTCH1	chr9:98209617	COSM1463798	c.3921del	Deletion	p.(Arg1308GlufsTer64)	Frameshift	23	Likely pathogenic
PTEN	chr10:89717770	rs121913289	c.800del	Deletion	p.(Lys267ArgfsTer9)	Frameshift	7	Pathogenic
PTPN21	chr14:88940113	rs778292578	c.2545del	Deletion	p.(Ile849LeufsTer9)	Frameshift	14	Likely pathogenic
PZP	chr12:9318694	rs150067154	c.2212C>T	Transition	p.(Arg738Ter)	Nonsense	18	Likely pathogenic
RBBP8	chr18:20548821	rs1183981352	c.306del	Deletion	p.(Lys102AsnfsTer20)	Frameshift	5	Likely pathogenic
REV3L	chr6:111709250		c.900dup	Duplication	p.(Phe301IlefsTer13)	Frameshift	9	Likely pathogenic
RNASEH2B	chr13:51530587	rs75254367	c.925del	Deletion	p.(Ile309LeufsTer26)	Frameshift	11	Likely pathogenic
ROR2	chr9:94487378		c.1397_1398del	Deletion	p.(Lys466ArgfsTer58)	Frameshift	9	Likely pathogenic
RSPH4A	chr6:116948995		c.1129del	Deletion	p.(Glu377LysfsTer11)	Frameshift	3	Likely pathogenic
SALL4	chr20:50400983	rs753320334	c.2983del	Deletion	p.(Val995PhefsTer14)	Frameshift	4	Likely pathogenic
SCN9A	chr2:167129256	rs4369876	c.2971G>T	Transversion	p.(Val991Leu)	Missense	17	Pathogenic
SCN9A	chr2:167133540	rs12478318	c.2794A>C	Transversion	p.(Met932Leu)	Missense	16	Pathogenic
SCN9A	chr2:167055992	rs1027061303	c.5124del	Deletion	p.(Val1709PhefsTer33)	Frameshift	27	Likely pathogenic
SEC63	chr6:108214755	rs752868449	c.1605del	Deletion	p.(Lys535AsnfsTer28)	Frameshift	16	Likely pathogenic
SEC63	chr6:108214773	rs777766787	c.1586dup	Duplication	p.(Lys530GlufsTer30)	Frameshift	16	Likely pathogenic
SERPINA6	chr14:94780642	rs113418909	c.344T>A	Transversion	p.(Leu115His)	Missense	2	Pathogenic
SETX	chr9:135202898	rs121434376	c.4087C>T	Transition	p.(Arg1363Ter)	Nonsense	10	Pathogenic
SLC9A9	chr3:143412150		c.534-1G>T	Transversion		Splice region	Intron 4	Likely pathogenic
SPTB	chr14:65260409		c.1972C>T	Transition	p.(Gln658Ter)	Nonsense	13	Likely pathogenic
STRA6	chr15:74501738	rs994494829	c.58C>T	Transition	p.(Gln20Ter)	nonsense	1	Likely pathogenic
SUCLG1	chr2:84658735		c.721_722del	Deletion	p.(Glu241AsnfsTer16)	Frameshift	7	Likely pathogenic
TAP2	chr6:32797242	rs771932254	c.1867C>T	Transition	p.(Arg623Ter)	nonsense	11	Likely pathogenic
TBC1D23	chr3:100039735	rs747637295	c.1947dup	Duplication	p.(His650ThrfsTer3)	Frameshift	18	Likely pathogenic
TBX1	chr22:19770492		c.1070del	Deletion	p.(Pro357GlnfsTer8)	Frameshift	9	Likely pathogenic
TCF7	chr5:133473765	COSV58655485	c.463del	Deletion	p.(His155ThrfsTer44)	Frameshift	4	Likely pathogenic
TCF7L2	chr10:114925317	rs745872748	c.1403del	Deletion	p.(Lys468SerfsTer23)	Frameshift	14	Likely pathogenic
TGFBR2	chr3:30691872	rs79375991	c.458del	Deletion	p.(Lys153SerfsTer35)	Frameshift	4	Likely pathogenic
TGM1	chr14:24718695	rs398122904	c.2278C>T	Transition	p.(Arg760Ter)	Nonsense	15	Likely pathogenic
TMPO	chr12:98921672	rs774744805	c.295del	Deletion	p.(Thr99LeufsTer12)	Frameshift	2	Likely pathogenic
TNXB	chr6:32035527	rs1457115836	c.6455del	Deletion	p.(Gly2152AlafsTer46)	Frameshift	18	Likely pathogenic
TOP1MT	chr8:144400187	rs780176693	c.1215+1del	Deletion		Splice region	Intron 9	Likely pathogenic
TPP2	chr13:103266498	COSV65750686	c.347del	Deletion	p.(Asn116MetfsTer54)	Frameshift	3	Likely pathogenic
TRPM1	chr15:31327754	rs1485132228	c.2746C>T	Transition	p.(Arg916Ter)	Nonsense	20	Likely pathogenic
TUBB2B	chr6:3225580	rs777598117	c.743C>T	Transition	p.(Ala248Val)	Missense	4	Likely pathogenic
CCN6	chr6:112389433	rs782739258	c.678dup	Duplication	p.(Cys227MetfsTer21)	Frameshift	4	Likely pathogenic
ZC3H3	chr8:144590070		c.1562-1G>A	Transition		Splice region	Intron 3	Likely pathogenic
ZFP90	chr16:68598463	rs747260313	c.1780del	Deletion	p.(Thr594ProfsTer57)	Frameshift	5	Likely pathogenic
ZNF469	chr16:88504202	COSV71258157	c.10247_10248dup	Duplication	p.(Arg3417GlyfsTer57)	Frameshift	2	Likely pathogenic

Gene	Location	rs/ COSMIC ID	Genomic consequence	Type	Protein alteration	Protein consequence	Exon	Clinical significance
ABCC6	chr16:16251531	rs58694313	c.3871G>A	Transition	p.(Ala1291Thr)	Missense	27	Pathogenic
ABCC6	chr16:16302586	rs72657698	c.793A>G	Transition	p.(Arg265Gly)	Missense	7	Pathogenic
ABCC6	chr16:16308294	rs192110266	c.487G>A	Transition	p.(Asp163Asn)	Missense	5	Pathogenic
ABCC6	chr16:16313545	rs55778939	c.346-6G>A	Transition		Splice region	Intron 3	Pathogenic
APC	chr5:112116513		c.559del	Deletion	p.(Arg187GlyfsTer18)	Frameshift	6	Likely pathogenic
C8B	chr1:57395210	rs757959349	c.1643G>A	Transition	p.(Trp548Ter)	Nonsense	12	Likely pathogenic
DOCK4	chr7:111368702		c.5529del	Deletion	p.(Leu1843PhefsTer27)	Frameshift	52	Likely pathogenic
ENO3	chr17:4859439	rs374679936	c.1067+1G>A	Transition		Splice region	Intron 9	Likely pathogenic
GALNS	chr16:88891261	rs118204437	c.1174C>T	Transition	p.(Arg392Cys)	Missense	12	Pathogenic
HLA-DRB1	chr6:32551885	rs200689965	c.370+1G>A	Transition		Splice region	Intron 2	Likely pathogenic
HYDIN	chr16:70896016	rs11337008	c.11712del	Deletion	p.(Gln3905ArgfsTer5)	Frameshift	69	Likely pathogenic
PPP2R2B	chr5:146460666	rs1424608676	c.5dup	Duplication	p.(Leu3AlafsTer5)	Frameshift	2	Likely pathogenic
PPP2R2B	chr5:146460668	rs1189662135	c.42G>A	Transition	p.(Trp14Ter)	Nonsense	2	Likely pathogenic
SCN9A	chr2:167129256	rs4369876	c.2971G>T	Transversion	p.(Val991Leu)	Missense	17	Pathogenic
SCN9A	chr2:167133540	rs12478318	c.2794A>C	Transversion	p.(Met932Leu)	Missense	16	Pathogenic
TMPRSS5	chr11:113567641	rs200381791	c.517del	Deletion	p.(Gln173ArgfsTer46)	Frameshift	6	Likely pathogenic

Gene	Location	rs / COSMIC ID	Genomic consequence	Type	Protein alteration	Protein consequence	Exon	Clinical significance
A4GALT	chr22:43089300		c.657_658del	Deletion	p.(Phe220ProfsTer62)	Frameshift	3	Likely pathogenic
ADAR	chr1:154574541	rs145588689	c.577C>G	Transversion	p.(Pro193Ala)	Missense	2	Pathogenic
ALDOB	chr9:104187213	rs145078268	c.911G>A	Transition	p.(Arg304Gln)	Missense	8	Likely pathogenic
CNGB1	chr16:57973498	rs373232101	c.1210-2A>G	Transition		Splice region	Intron 14	Likely pathogenic
COL4A3	chr2:228118852		c.791del	Deletion	p.(Lys264ArgfsTer59)	Frameshift	14	Likely pathogenic
EYS	chr6:64431310		c.8679_8680del	Deletion	p.(Asp2894LeufsTer2)	Frameshift	44	Likely pathogenic
FBN1	chr15:48782210	rs397514558	c.2920C>T	Transition	p.(Arg974Cys)	Missense	25	Pathogenic
HNF1B	chr17:36093549		c.809+1G>A	Transition		Splice region	Intron 3	Likely pathogenic
KIR2DL4	chr19:55324674	rs11371265	c.810dup	Duplication	p.(Met271AsnfsTer108)	Frameshift	6	Likely pathogenic
KRAS	chr12:25398284	rs121913529	c.35G>A	Transition	p.(Gly12Asp)	Missense	2	Pathogenic
MC1R	chr16:89986117	rs1805007	c.451C>T	Transition	p.(Arg151Cys)	Missense	1	Pathogenic
PKHD1	chr6:51611651	rs148932323	c.9866G>T	Transversion	p.(Ser3289Ile)	Missense	59	Likely pathogenic
PRF1	chr10:72360387	rs35947132	c.272C>T	Transition	p.(Ala91Val)	Missense	2	Pathogenic
PRKN	chr6:162864358	rs754809877	c.155del	Deletion	p.(Asn52MetfsTer29)	Frameshift	2	Pathogenic
RET	chr10:43620335	rs17158558	c.2944C>T	Transition	p.(Arg982Cys)	Missense	18	Pathogenic
ROPN1L	chr5:10448375	rs41280363	c.135T>A	Transversion	p.(Tyr45Ter)	Nonsense	2	Likely pathogenic
SARDH	chr9:136595263	rs1463674467	c.733_736dup	Duplication	p.(Asp246GlyfsTer2)	Frameshift	5	Likely pathogenic
SCO2	chr22:50962500	rs145100473	c.341G>A	Transition	p.(Arg114His)	Missense	2	Pathogenic
TRPV4	chr12:110240859	rs187864727	c.649G>T	Transversion	p.(Ala217Ser)	Missense	4	Pathogenic

Gene	Location	rs /COSMIC ID	Genomic consequence	Type	Protein alteration	Protein consequence	Exon	Clinical significance
CD109	chr6:74520836	rs1431635443	c.3675_3676del	Deletion	p.(His1225GlnfsTer19)	Frameshift	*28*	Likely pathogenic
HYDIN	chr16:70896016	rs11337008	c.11712del	Deletion	p.(Gln3905ArgfsTer5)	Frameshift	69	Likely pathogenic
KIR2DL4	chr19:55324674	rs11371265	c.810dup	Duplication	p.(Met271AsnfsTer108)	Frameshift	6	Likely pathogenic
KRAS	chr12:25398284	rs121913529	c.35G>A	Transition	p.(Gly12Asp)	Missense	2	Pathogenic
MS4A2	chr11:59860986	rs1235778884	c.492C>A	Transversion	p.(Cys164Ter)	Nonsense	5	Likely pathogenic
MUC6	chr11:1027390	rs200217410	c.2109C>A	Transversion	p.(Cys703Ter)	Nonsense	17	Likely pathogenic
PAFAH1B3	chr19:42804151	rs775116993	c.379C>T	Transition	p.(Arg127Ter)	Nonsense	5	Likely pathogenic
PIK3CA	chr3:178916876	rs121913287	c.263G>A	Transition	p.(Arg88Gln)	Missense	2	Likely pathogenic
PTCD1	chr7:99017730	rs779939675	c.1963C>T	Transition	p.(Arg655Ter)	Nonsense	8	Likely pathogenic
TP53	chr17:7577114	rs863224451	c.824G>T	Transversion	p.(Cys275Phe)	Missense	8	Likely pathogenic
TRPV4	chr12:110240859	rs187864727	c.649G>T	Transversion	p.(Ala217Ser)	Missense	4	Pathogenic
