# Supplementary figures and images for: Molecular Profiling of Tumor Tissue in Mexican Patients with Colorectal Cancer
Source: Curr Issues Mol Biol. 2022 Aug 20;44(8):3770–8. doi: 10.3390/cimb44080258 (PMC9406459; doi:10.3390/cimb44080258)

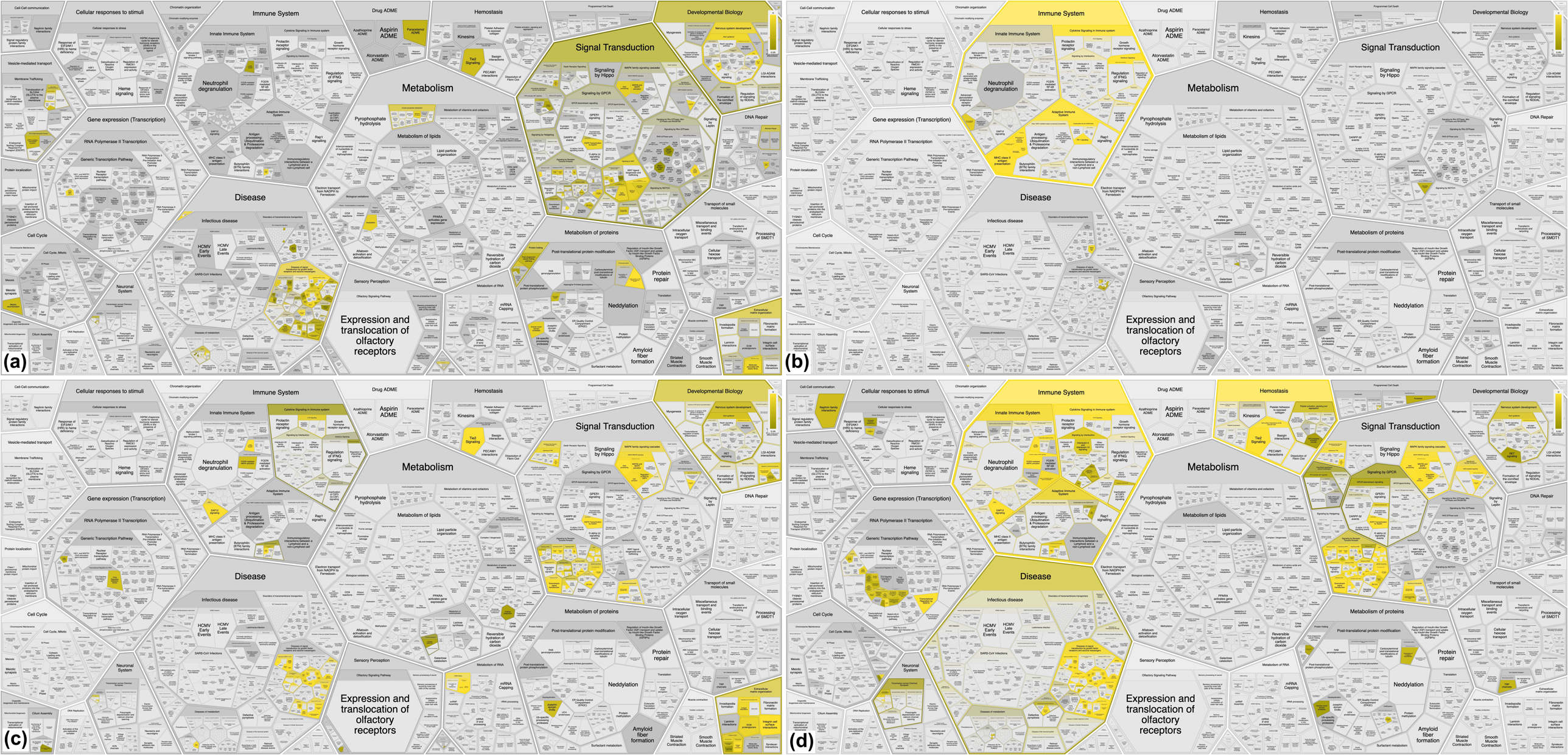

Supplement: Supplementary file 1 [file cimb-44-00258-s001.zip › Figure S1-Flores-López BA.jpg]
